# Wheat Protein-Induced Enterocolitis Syndrome Complicated With Wheezing: An Approach to Diagnosis and Wheat Reintroduction

**DOI:** 10.7759/cureus.17141

**Published:** 2021-08-13

**Authors:** Miwa S Shinohara, Ryuji Nino, Takashi Higaki, Eiichi Ishii

**Affiliations:** 1 Medical Sciences in Clinical Investigation, Harvard Medical School, Boston, USA; 2 Department of Pediatrics, Juntendo University Faculty of Medicine, Tokyo, JPN; 3 Department of Pediatrics, Ehime University Hospital, Toon, JPN; 4 Department of Pediatrics, Ehime Prefectural Imabari Hospital, Imabari, JPN; 5 Department of Pediatrics, Ehime University Faculty of Medicine, Toon, JPN; 6 Center for Transition to Adult Congenital Heart Disease, Ehime University Hospital, Toon, JPN; 7 Department of Pediatrics, Imabari City Medical Association Municipal Hospital, Imabari, JPN

**Keywords:** reintroduction, protein-induced enterocolitis syndrome, wheezing, wheat, oral food challenge

## Abstract

An 11-month-old Japanese girl was diagnosed with food protein-induced enterocolitis syndrome (FPIES) after presenting with vomiting approximately two hours after wheat intake. She showed positive results on the first wheat oral food challenge (OFC) at nine months of age, although serum wheat- and ω-5 gliadin-specific immunoglobulin E (IgE) were not detected. The second wheat OFC, performed at age 13 months, induced wheezing (usually an IgE-mediated symptom) 4.5 hours after ingestion, probably owing to gastroesophageal reflux caused by repetitive vomiting. The third wheat OFC, performed at age 25 months, did not trigger reactions. Therefore, gradual low-dose wheat was reintroduced at home. The fourth wheat OFC performed at age 30 months induced no response either; thus, the patient was deemed to have developed tolerance to wheat. This case report, therefore, demonstrates that careful assessment of natural history and physician-supervised OFCs are necessary for adequate diagnosis and the successful management of reintroduction in wheat-induced FPIES.

## Introduction

Background

Food protein-induced enterocolitis syndrome (FPIES) caused by solid food is a type of non-immunoglobulin E (IgE)-mediated gastrointestinal food hypersensitivity. FPIES manifests as profuse, repetitive vomiting, often with diarrhea, within two to six hours of ingesting trigger foods, and failure to thrive if chronic [1]. FPIES is most commonly elicited by cow’s milk (CM) and soy (CM-/soy-induced FPIES). However, rice, oats, and other solid foods may elicit FPIES (solid food-induced FPIES) [2-4].

Geographic variability exists among the offending foods [5]. Although the majority of children will outgrow FPIES by approximately three to four years of age, the natural history of FPIES varies widely and depends on the study population, causative foods, and coexisting atopic diseases [6]. Avoidance of the offending food is currently the only specific treatment for FPIES. A physician-supervised oral food challenge (OFC) can be used to establish the diagnosis and to determine the feasibility of reintroducing the offending food [1,7]. Moreover, as reintroduction failure is common in children after negative OFCs, an adequate follow-up strategy is needed.

Wheat-induced FPIES cases are rarely reported [7] because, in Japan, gastrointestinal food allergies affect 0.5% of children and the prevalence of wheat-induced FPIES at age 1.5 years is 0.08% of non-IgE mediated food allergies [8]. Moreover, in wheat-induced FPIES, the only available diagnosis and follow-up management tests are the physician-supervised OFC [9-10]. Here, we report the case of a Japanese female infant with wheat-induced FPIES whose condition was successfully diagnosed and managed using physician-supervised OFCs and gradual low-dose reintroduction of wheat.

## Case presentation

A full-term 11-month-old Japanese girl with no abnormalities during the gestational period or at delivery was transferred to our hospital. She was initially breastfed and supplemented with a cow’s milk (CM)-based formula. She had a strong family history of allergy; her father had a history of food allergy (FA) to eggs during his childhood, and her mother had had atopic dermatitis (AD) since childhood. The patient was an only child. At age eight months, she could eat one spoonful of boiled egg white several times but had never eaten soy. At ages five to six months, she ate a few spoonsful of wheat noodles once and a few pieces of bread a couple of times, without showing any clinical symptoms. However, at seven, eight, and nine months of age, consumption of the wheat noodles led to three episodes of vomiting after approximately two hours. At nine months of age, blood examinations at the pediatric department showed normal eosinophil levels in her peripheral blood, with no eosinophils detected in her stool. Serum food-specific IgE antibodies against wheat and ω-5 gliadin were not detected, and the vomiting episodes were thus thought to be a result of non-IgE-mediated FA. In contrast, serum food-specific IgE antibodies for egg white, egg yellow, CM, and soy were detected (Table 1). The pediatrician thus recommended that wheat, egg, and soybean be avoided. Following the recommendation, the vomiting after wheat protein ingestion disappeared. The first open OFC to wheat protein (0.2 g/kg, gram of the food protein per kilogram of body weight) resulted in repeated vomiting (>10 times), lethargy, and pallor two hours after ingestion of wheat protein. The patient was diagnosed with a late reaction to wheat FA and was treated with intravenous corticosteroid (hydrocortisone sodium succinate 5 mg/kg). The pediatrician recommended continued avoidance of wheat, egg, and soybean. The vomiting episodes after food ingestion ceased. At 11 months of age, the patient relocated and was referred to our department. Our initial physical examination showed a failure to thrive since eight months of age (body weight (BW) -1.0 SD), dry skin since five months of age, and moderate AD on her body. However, she had a good appetite and did not have constipation, abdominal pain, signs of irritable bowel syndrome, dyspepsia, malabsorption, iron deficiency anemia, or oral aphthae.

**Table 1:** Physical symptoms and laboratory results at the initial physical examination and after the OFC with wheat noodles. *Time when the physical symptom was developed by OFC. **Data (institutional reference range) OFC, oral food challenge; WBC, white blood cell; Seg, segmented neutrophil; band, band neutrophil; CRP, C-reactive protein; TP, total protein; Alb, albumin; TARC, thymus and activation-regulated chemokine; SPT, skin prick test; LST, lymphocyte stimulation test; S.I., stimulation index; HRTs, basophil histamine release tests

OFC to wheat				First			Second				Third				Fourth		Reference range
Patient's age (months)				9			13				25				30		
Noodle (wheat protein, mg/kg)				0.5 (0.2)			0.6 (0.2)				0.6 (0.2)				8.0 (2.7)		
Time after ingestion (hours)				0	2		0	3-5	22		0	6	22		0	6	
Physical symptom*																	
	Hypothermia			-	-		-	-	-		-	-	-		-	-	
	Hypotension			-	-		-	-	-		-	-	-		-	-	
	Vomiting			-	+		-	+	-		-	-	-		-	-	
	Lethargy			-	+		-	+	-		-	-	-		-	-	
	Pallor			-	-		-	-	-		-	-	-		-	-	
	Diarrhea			-	-		-	-	-		-	-	-		-	-	
	Wheeze			-	-		-	+	-		-	-	-		-	-	
	Stool examinations																
		Hemoglobin (ng/ml)						-			-						-
		Transferrin (ng/ml)						-			-						-
		Eosinophil (%)		-	-		-	-			-						-
Laboratory results**																	
	Complete blood cell count																
		WBC (/mm^3^)		16300			8200	7300	9000		8900	8200	6000		9000	12900	(3500 - 9100)
			Seg (%)	56.0			13.5	34.0	33.0		35.0	41.6	29.5		47.8	42.5	(0.0 - 10.0)
			Band (%)	6.0			0.5	3.0	1.0		0.0	0.0	0.0		0.0	0.5	(39.1 - 72.5)
			Monocyte (%)	5.1			1.5	1.0	4.0		6.0	9.4	5.0		2.9	5.0	(3.1 - 8.7)
			Eosinophil (%)	0.1			1.5	0.0	0.0		2.0	0.5	3.0		1.4	2.0	(0.0 - 8.0)
			Basophil (%)	0.1			0.0	0.0	1.0		1.0	0.2	0.0		0.3	0.0	(0.0 - 1.9)
			Lymphocyte (%)	34.0			83.0	62.0	60.0		56.0	40.6	61.0		47.6	49.5	(20.0 - 50.6)
	Neutrophil count (/mm3)							+1553	+1912			+296	-1345			+1245	
	Serum chemistry																
		CRP (mg/dL)		0.02			<0.01	0.70	1.49		0.02	0.02	0.01		0.01	0.01	(0.00 - 0.20)
		TP (g/dL)					6.8				6.7				6.0		(6.6 - 8.2)
		Alb (g/dL)					4.7				4.3				3.8		(3.9 - 4.9)
	TARC (pg/ml)						1152				1003				737		(<1367)
	Serum IgE																
		Total IgE (IU/mL)		162			104				174						(< 165)
		Wheat (UA/mL)		0.02			<0.10				<0.10						(<0.35)
		ω-5 gliadin (UA/mL)		0.01			<0.10				<0.10						(<0.35)
		Rye (UA/mL)					<0.10										
		Barley (UA/mL)					<0.10										
		Oat (UA/mL)					<0.10										
		Egg white (UA/mL)		19.20			13.30				2.11						(<0.35)
		Egg yellow (UA/mL)		2.03			1.47				0.24						(<0.35)
		Ovomucoid (UA/mL)		24.40			12.60				1.47						(<0.35)
		Soy (UA/mL)		1.45			0.48										(<0.35)
	SPTs																
		Saline					-				-				-		
		Wheat					-				-				-		
		Egg white					+				+				+		
	LST (SRL)																
		Wheat (cpm)					683				890				1188		
		S.I. (%)					154				114				1015		(≦180)
		Control (cpm)					441				775				117		
	HRTs																
		Wheat (class)					0										
		Egg white (class)					0										
		Soy (class)					0										
		Rice (class)					0										
		Milk (class)					0										
		Total histamine release (nmol/L)					337.3										
		Non-specific histamine release (nmol/L)					42.8										

Investigations

Laboratory findings revealed neither increased peripheral eosinophils nor eosinophils in the stool. Skin prick tests (SPTs) for wheat and serum food-specific IgE antibodies against wheat and ω-5 gliadin were not detected. The wheat-specific lymphocyte stimulation test (LST) (SRL, Tokyo, Japan) was negative. To determine her FA condition, the second OFC to wheat protein at 0.2 g/kg was performed at 13 months of age. Three hours after ingestion, she showed three episodes of vomiting. After 4.5 hours of ingestion, she developed wheezing (usually an IgE-mediated symptom), confirmed by two pediatricians (Table 1). She was treated with intravenous hydration and 5 mg/kg of hydrocortisone sodium phosphate. We recommended that she continues avoiding wheat.

Differential diagnosis

According to the international consensus guidelines for FPIES [11-12] diagnosis of FPIES is clinical, based on typical characteristic signs and symptoms. These signs and symptoms usually improve after withdrawal of the suspected trigger food, and by repeated physician-supervised OFCs with a causative food dose of 0.06-0.6 g/kg (usually 0.3 g/kg). In our case, the onset of solid food-induced FPIES was observed at a higher age (6-12 months) than the age of presentation for CM-/soy-induced FPIES (<6 months) [12]. Additionally, a confirmatory OFC is considered unnecessary when the typical symptoms occur within two to four hours after food ingestion (particularly more than once), and the child remains well if the food is eliminated from the diet [13]. In our case, the patient had shown three episodes of wheat-noodle ingestion at home since she was seven months old, and the first wheat OFC at nine months was positive. Additionally, the first OFC to wheat-induced typical FPIES symptoms (repetitive vomiting, pallor, and lethargy) within two hours of ingestion. The international consensus guidelines for the diagnosis and management of FPIES [12] relies on the major and minor criteria in making a diagnosis of FPIES in patients with a history of possible or confirmed FPIES. The major criteria include vomiting in the one- to four-hour period after ingestion of the suspect food and the absence of classic IgE-mediated allergic skin or respiratory symptoms. The minor criteria include lethargy, pallor, diarrhea five to 10 h after food ingestion, hypotension, hypothermia, and increased neutrophil count of >1500 above the baseline. The OFC is diagnostic for FPIES if the major criterion and >2 minor criteria are met; accordingly, our patient was diagnosed with wheat-induced FPIES.

 A biopsy is needed for histologic confirmation of food-protein-induced enteropathy. However, it is usually not indicated in patients with FPIES presenting with acute symptoms or in patients with food-protein-induced allergic proctocolitis [14]. According to the guidelines [11-12], the clinician needs to exclude other potential causes and use OFCs to help confirm the diagnosis if the history is unclear and there is a favorable risk/benefit ratio. Even in patients with chronic FPIES where history alone is inadequate to make a diagnosis, a trial of food elimination followed by supervised OFCs to potential food triggers might be required. In select patients, endoscopy and biopsy might be warranted to exclude other causes. Our patient showed a failure to thrive just after onset and resolution of wheat FPIES, and no gastric tract symptoms, such as constipation, abdominal pain, signs of irritable bowel syndrome, dyspepsia, malabsorption, iron deficiency anemia, and oral aphthae, were observed. Celiac disease (CD) was therefore unlikely, and we decided to conduct periodic follow-ups and provide nutritional guidance and OFCs. Regarding chronic FPIES, the previous pediatrician recommended continued avoidance of wheat, egg, and soybean, thus, in her first visit to our university hospital, a nutritionist checked her food intake. The patient took enough energy almost from vegetables, rice, and sugar but not from meat and fish. Because vegetable rarely causes FPIES, vomiting appeared only when she ate wheat all through her clinical course, and her family member looks like the same growth pattern. Thus, I judged the possibility of chronic FPIES that other foods induce is very low.

Diagnosis of acute FPIES is usually delayed because of the absence of classic allergic symptoms and the lack of timely obtainable biomarkers. Measurements of food-specific serum IgE levels usually take at least several days in clinical institutes. In this case, the second wheat OFC-induced wheezing lung sounds (a classical IgE-mediated allergic respiratory symptom). Based on the negative results of the SPT to wheat, the second wheat OFC, and previous episodes of vomiting restricted exclusively to wheat intake, we considered that the wheezing sounds might have been caused by the aspiration of food contents due to gastroesophageal reflux disease. Thus, we did not perform invasive 24-hour pH monitoring. Additionally, we observed an increased neutrophil count of ≥ 1,500 above the baseline, at five and 22 hours after ingestion. Several days after the second wheat OFC, we received negative tests results for serum wheat- and ω-5 gliadin-specific IgE at the second wheat OFC. Although we did not examine serum histamine, serum tryptase, and urine N-methylhistamine due to the unavailability of the patient’s serum and urine, the symptoms induced by the second wheat OFC met at least three minor criteria of the international consensus guidelines for FPIES [11].

Treatment

At age 25 months, the third OFC for wheat protein at 0.2 g/kg did not induce any clinical symptoms (Table 1). Due to the negative results, a pediatric allergy specialist recommended a protocol for a gradual low-dose reintroduction of wheat. Our protocol was designed to provide a total amount of 1.0 g/kg/day of wheat protein in 19 weeks and included an induction phase (dose of wheat protein ≤0.2 g/kg/day) and an escalation phase (the dose of wheat protein >0.2 g/kg/day). These phases were separated by the dose of protein at the third OFC (0.2 g/kg), which the patient was supposed to consume without manifesting clinical symptoms. The gradual low-dose reintroduction of wheat was started after the establishment of an emergency network, which included the availability of a pediatrician within 20 minutes of request. For the first week, she ingested 0.1 g/kg/day of wheat protein once on a weekday morning at home. During the first seven weeks of the induction phase, the frequency of wheat-protein ingestion was increased by one day weekly, until a frequency of once per day, seven days per week was achieved. Then, in the escalation phase, the dose of wheat was increased to 1.0 g/kg/day of wheat protein once weekly, and the increasing speed of wheat consumption was spontaneously decreased weekly (Table 2). During both the induction and the escalation phases, no adverse symptoms were observed.

**Table 2:** Reintroduction schedule for wheat

Phase (week)	Frequency			Increased dose per one ingestion			Cumulative dose	
	(time/day)	(day/week)		(g/day)	(%)		(g/day)	(g/kg/day*)
Induction Phase (≦0.2 g/kg/day*)								
1	1	1		0.0	0.0		1.3	0.1
2	1	2		0.0	0.0		1.3	0.1
3	1	3		0.0	0.0		1.3	0.1
4	1	4		0.0	0.0		1.3	0.1
5	1	5		0.0	0.0		1.3	0.1
6	1	6		0.0	0.0		1.3	0.1
7	1	7		0.0	0.0		1.3	0.1
8	1	7		0.7	54		2.0	0.2
9	1	7		1.0	50		3.0	0.2
Escalation Phase (>0.2 g/kg/day*)								
10	1	7		1.0	33		4.0	0.3
11	1	7		1.0	25		5.0	0.4
12	1	7		1.0	20		6.0	0.5
13	1	7		1.0	17		7.0	0.5
14	1	7		1.0	14		8.0	0.6
15	1	7		1.0	13		9.0	0.7
16	1	7		1.0	11		10.0	0.8
17	1	7		1.0	10		11.0	0.9
18	1	7		1.0	9		12.0	0.9
19	1	7		1.0	8		13.0	1.0
20	1	7		1.0	8		14.0	1.1

Outcome and follow-up

At 30 months of age, the patient could ingest up to 14.0 g/day (1.1 g/kg/day) of wheat protein without showing adverse reactions. This implied that the threshold dose of wheat that she could eat might have increased or the disease spontaneously resolved following its natural history. In the OFCs to wheat protein, we used “udon noodles,” for which the common dose in one meal in Japan is 200 g (wheat protein 66.7 g) for an adult and 100 g (wheat protein 33.3 g) for a 3-year-old child. Thus, the dose of wheat protein in the fourth OFC with udon noodles (body weight 12.3 kg) was 33.3 g (wheat protein 2.7 g/Kg). The fourth OFC to wheat protein at 2.7 g/kg induced no symptoms and suggested that she had developed tolerance to wheat. She has been able to eat wheat freely from age 30 months to at least until 44 months of age.

During all the OFCs, hypotension, hypothermia, diarrhea, and eosinophils in the stool were not reported (Table 1). Throughout the entire clinical course, SPTs for wheat and serum food-specific IgE antibodies for wheat and ω-5 gliadin were negative. Her failure to thrive since she was seven months old improved spontaneously after 13 months of age. During this period, energy intake was sufficient for her age, and growth hormone levels were within the normal range. The patient’s AD was also successfully treated with an emollient and mild steroid ointment since she was 11 months old and has been the only treatment administered to her from 12 to 35 months of age.

## Discussion

We report a case of wheat-induced FPIES in a Japanese female infant, which was detected at seven months of age and was successfully reversed with the gradual, low-dose reintroduction of wheat protein from 25 months of age. Although the possibilities - this approach caused tolerance, the disease resolved following the natural history, or the OFCs were false negative, remained, tolerance was confirmed at 33 months after these physician-supervised OFCs.

The natural course of wheat-induced FPIES remains unclear. According to the guideline, diagnosis of FPIES is primarily based on the clinical history of characteristic signs and symptoms, with improvement being observed after withdrawal of the suspected trigger food [11-12]. In our case, wheat OFCs were performed at first contact to confirm the diagnosis and for documentation of resolution. OFCs in patients with FPIES should be supervised by a physician/pediatrician. Home OFCs against a suspected trigger are discouraged [11] because up to 50% of positive OFCs could result in hypovolemic shock, which requires treatment with intravenous fluids. A single dose of intravenous steroids can decrease presumed cell-mediated inflammation, although no studies support this recommendation [15]. In this case, we used intravenous hydration and steroids.

Resolution of FPIES varies widely, depending on the study population, the causative foods, and any coexisting atopic disease. Between 2% and 20% of children with increased serum IgE in response to their food trigger (so-called atypical FPIES) exhibit slow resolution [7]. Additionally, desensitization procedures and oral immunotherapy to FPIES have not been established and reintroduction protocol is important in the long-term management of FPIES (Table 2). To determine the threshold amount of wheat ingestion or acquisition of tolerance, we performed the fourth wheat OFC, which required the patient to consume approximately 2.7 g/kg of wheat protein. This induced no symptoms at 30 months of age.

The wheat-specific LST might be a useful tool for diagnosing delayed-type food allergies, especially in milk-induced FPIES, which manifests as gastrointestinal symptoms but not as skin symptoms [16]. Moreover, there are no in vitro tests capable of confirming FPIES, and the precise immune mechanisms involved are unclear [17]. In this particular case, the commercial DLST to wheat (SRL) was not useful in the diagnosis of wheat-induced FPIES.

For the differential diagnosis, CD develops in genetically susceptible individuals who, in response to unclear environmental triggers, develop gastrointestinal symptoms such as chronic or intermittent diarrhea, chronic constipation not responding to usual treatment, and chronic abdominal pain. Other intestinal symptoms of CD include a distended abdomen, recurrent nausea, and recurrent vomiting. Extra-intestinal symptoms, such as weight loss, failure-to-thrive, irritability, chronic fatigue, chronic iron deficiency, recurrent aphthous, neuropathy, and abnormal liver biochemistry may be present [18-19]. Even in patients with symptoms-suspected CD, the European Society for Paediatric Gastroenterology, Hepatology and Nutrition has revised its recommendations to include a diagnostic algorithm that includes sequential serological testing and human leukocyte antigen genotyping for symptomatic children, which would enable a diagnosis of celiac disease to be made in the absence of a confirmatory intestinal biopsy [19-20].

## Conclusions

In conclusion, this case illustrates that detailed clinical history and repeated physician-supervised OFCs are necessary for adequate diagnosis and successful management, through reintroduction, in wheat-induced FPIES. For the diagnosis and treatment of FPIES, accurate and promptly obtainable diagnostic biomarkers are required as soon as possible.
